# Synthesis and physicochemical characterization of bovine lactoferrin supersaturated complex with iron (III) ions

**DOI:** 10.1038/s41598-022-15814-2

**Published:** 2022-07-26

**Authors:** Oleksandra Pryshchepa, Katarzyna Rafińska, Adrian Gołębiowski, Mateusz Sugajski, Gulyaim Sagandykova, Piotr Madajski, Bogusław Buszewski, Paweł Pomastowski

**Affiliations:** 1 Centre for Modern Interdisciplinary Technologies, Nicolaus Copernicus University in Torun, Wileńska 4, 87-100 Torun, Poland; 2 Department of Environmental Chemistry and Bioanalytics, Faculty of Chemistry, Nicolaus Copernicus University in Torun, Gagarina 7, 87-100 Torun, Poland; 3 Department of Chemistry of Materials Adsorption and Catalysis, Faculty of Chemistry, Nicolaus Copernicus University in Torun, Gagarina 7, 87-100 Torun, Poland

**Keywords:** Bioinorganic chemistry, Metals, Proteins, Metals, Proteins

## Abstract

The aim of the study was to investigate the process of Fe^3+^ binding to bLTF. Moreover, the physicochemical characterization of the respective supersaturated complex was studied. The knowledge should be important for the description of processes that may take place in dairy products fortified with iron. Additionally, the synthesized complex can be utilized as a dietary supplement for the treatment of iron deficiency anemia (IDA). Finally, it was shown that formation of supersaturated iron-protein structures which include LTF often accompanies development of neurodegenerative diseases such as Alzheimer or Parkinson. Thus, the study can reveal some aspects of its pathogenesis process. The methodology of the investigation comprised the utilization of batch sorption study and applying Freundlich and Langmuir models. The complex also was characterized by numerous techniques: spectrometric (ICP-MS), spectroscopic (UV–Vis, ATR-FTIR), electron microscopy (TEM–EDX), SDS-PAGE. Based on obtained results the potential mechanisms of iron interaction with protein were described. Moreover, the molecular docking was applied to visualize possible metal binding sites. The respective complex contains ≈ 33.0 mg/g of iron which is nearly 50 Fe^3+^ per one protein molecule. The cytotoxicity of the obtained complex was evaluated by MTT reduction and LDH release assays on Caco-2 and nL929 cell lines.

## Introduction

Lately, big attention is paid to the production of so-called “functional food”. The term has been derived from knowledge about the link between nutrition and health. The concept implies the production of food with beneficial effects on health by enrichment with the biologically active components, e.g. vitamins, microelements, probiotic bacteria, etc.^1,2^. Consumption of dairy products by humans is known from ancient times. Thus, substances derived from milk are considered safe and healthy. Milk proteins, and in particular lactoferrin (LTF), are the compounds that show the widest spectrum of biological activity in the organism^1,3,4^. Thus, the production of new biologically active substances based on milk proteins attracts much attention as a part of “functional food” development^1,2^.

Iron deficiency anemia (IDA) is an issue of worldwide importance. More than 1.68 billion people are affected by IDA globally^5^. The importance of iron in the organism cannot be overestimated, as it makes up heme of hemoglobin and myoglobin which take part in oxygen transport and storage. Moreover, iron also is part of the cytochromes, peroxidases, nitric oxide synthases, and other enzymes that are involved in the processes of biosynthesis and energy production, detoxification, host defense, cellular signaling, etc.^6^. Iron in the human body can be absorbed in two forms, namely heme- (from meat) and non-heme. The non-heme iron is mainly absorbed in reduced Fe^2+^ form through non-specific transport with involvement of divalent metal ion transporter-1, while usually enters to the body in oxidized form Fe^3+^^5^. However, Fe^3+^ bioavailability is highly dependent on its solubility meaning that it needs to be bound to proteins or other hydrophilic chelators^6^.

For IDA treatment oral preparations of Fe^2+^ (e.g., fumarate, or sulfate) are often utilized, which can lead to gastrointestinal side effects. Instead, the utilization of bovine LTF (bLTF) has shown to have the same effect in improving hematological parameters accompanying IDA (serum ferritin and iron levels) as ferrous sulfate salt, but with lower gastrointestinal side effects^7^. The bLTF impact can be connected to its structure, which is highly homologous to the human LTF^3^ and hence bLTF, as well as its peptides, can interact with specific intestine lactoferrin receptors (human intelectin)^8^. In turn, it was shown that both native human LTF and its fragments can take part in directed transport of the Fe^3+^ to the brush-border membrane by binding to lactoferrin receptors, which can be considered as a specific carrier-mediated iron uptake mechanism^7,9^. Moreover, the effect of LTF can be also connected to the modulation of action of iron regulatory proteins, which promotes iron efflux from cells to the blood. Additionally, high affinity of iron to bLTF may enhance the Fe^3+^ solubility in the gastrointestinal tract^7^.

Literature data also reveals that microelements, in particular iron, in form of complexes with proteins, i.e., so-called proteinates, has higher bioavailability than inorganic salts^10–12^. There are several studies and patents presenting the synthesis of respective complexes based on the milk and whey proteins. For instance, Dalev (1993) in its work presents the technology for the preparation of iron-protein complexes based on the whey^13^. The procedure, among others, implies heat denaturation of all proteins to obtain precipitate. However, such procedure leads to the loss of the biological activity of the proteins. Another example is the synthesis of succinylated caseinate iron complex^14,15^. The succinylation is a process that aimed to enrich the protein with carboxylic groups and thus to improve the functional properties of proteins, among others, better emulsifying, foaming, gelation but also to increase the iron-binding ability^16^. Thus, the possibility to obtain iron-rich complexes based on unmodified proteins is of great interest and bLTF seems to be the most suitable for such preparations.

bLTF is the protein belonging to the transferrin family, which are the non-heme iron-binding proteins. Transferrins typically consist of a single polypeptide chain comprised of 670–700 amino acids, which form two globular lobes (N- and C-lobes) with one iron-binding site each^3,4,17^.The meaning „iron saturated” is usually utilized for the description of bLTF that has both binding sites filled with iron and called holo-lactoferrin. However, it was shown that bLTF can bind much more iron than can be predicted by its structure which should result information of supersaturated complex. The existence of such complexes can be a reason of beneficial effect on the non-heme iron bioavalability from food and dietary supplements which is poorly lightened in the literature^18,19^. However, it is also noteworthy to mention, that LTF has been indicated to take part in Alzheimer’s disease due to its presence in senile plaque, while the formation of amyloids is often connected to iron overload. One of the important aspects is that senile plaque can include iron-oxide magnetic nanoparticles in its core^20^, which shows the negative aspects of the formation of supersaturated Fe-protein complexes. Thus, there is the need for the comprehensive study of the formation mechanisms of the Fe-bLTF complex as well as its physicochemical and biological properties. Such studies may provide knowledge about the fate of iron in products fortified with microelements. Moreover, the corresponding complex can be utilized as dietary supplements and thus can be a solution in the treatment of IDA. Still, the studies can also provide additional information about mechanisms of iron toxicity, among others on the development of neurodegenerative diseases.

Hence, the work aimed to synthesize the supersaturated complex of bLTF with Fe^3+^. Additionally, the mechanisms which are involved in the formation of the respective complexes were investigated. The respective knowledge can be useful for the prediction of bLTF behavior in iron-rich solutions, among others in nutrient formulas. The synthesized complex was also subjected to physicochemical characterization. Moreover, the stability and cytotoxicity of the synthesized complex were evaluated.

## Results and discussion

### ***The batch sorption study of Fe***^***3***+^***interaction with bLTF***

**Figure 1:**
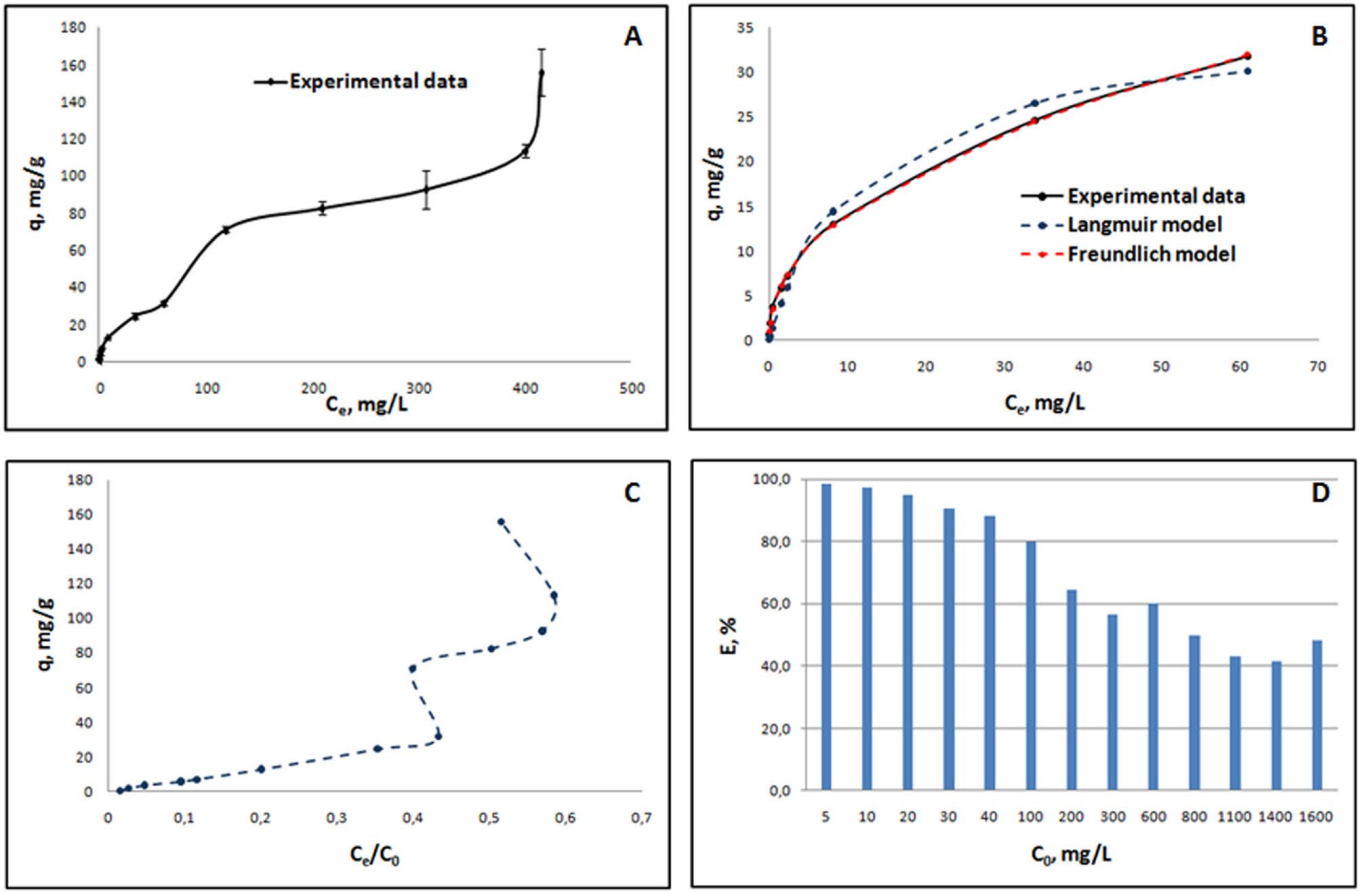
Batch isotherm study of Fe^3+^ sorption onto bLTF: (**A**) isotherm of Fe^3+^ adsorption onto bLTF for entire concentration range, (**B**) the fit of first sorption step to Langmuir and Freundlich models, (**C**) modified BET isotherm, which presents adsorption as a function of a ratio C_e_/C_0_, (**D**) bar chart of a sorption efficiency of bLTF depending on the initial concentration of Fe^3+^.

The isotherm study was performed to investigate the maximum sorption capacity of bLTF and to predict possible processes which may take place during the interaction. Figure 1A. presents the results of the batch isotherm study for the entire range of utilized Fe^3+^ concentrations. As can be seen from the experimental data the process has heterogeneous character—the formation of “several adsorption layers” can be predicted. Moreover, the obtained isotherm belongs to IV/VI type according to IUPAC classification, meaning that during the interaction the layer-by-layer adsorption occurs^21^. Langmuir and Freundlich isotherm models are often utilized for the description of the sorption processes. Both of the mentioned models are simple and were shown to be useful for the description of the processes of protein interaction with such metals as Ag^+^ and Zn^2+^^22,23^. However, the models can be applied only to isotherm type I, which corresponds to the first step of the IV/VI type isotherm^21^.

Previous studies^22,23^ have shown that utilization of the modified BET isotherm proposed by Sprynskyy et al.^24^ was useful for the description of the multilayer adsorption process and enabled to distinguish concentration ranges for the formation of each subsequent layer. In the present study, such an approach has shown that in the entire concentration range the formation of three adsorption layers on the surface of the bLTF can be predicted (Fig. 1C). The first layer is formed when the Fe^3+^ solutions of initial concentration up to 300 mg/L was utilized, which can be seen more evidently from the bar chart of bLTF sorption efficiency (Fig. 1D). The formation of the second layer was observed for the Fe^3+^ concentrations between 600 and 1400 mg/L. Third layer formation begins at a Fe^3+^ concentration of 1600 mg/L. It is noteworthy to mention, that classical isotherm models were developed for the description of surface sorption where the adsorbate in second adsorption layer is characterized by lower energy of adsorption. In case of protein interactions, the formation of multiple “adsorption layers” should indicate the interaction of different nature, i. e. the formation of nanoparticles, metal chelation, or electrostatic interactions. Interestingly, before the study, the physicochemical characterization of the protein was performed. The results are presented in our previous work^25^. It turned out that purchased from Sigma-Aldrich bLTF has both metal-binding sites filled with iron (the Fe^3+^ content in the unmodified protein accounts 1.45 ± 0.06 mg/g). Thus, it can be argued that iron-binding occurs outside the previously described metal-binding sites.

Further, the Freundlich and Langmuir models were applied to the outlined first adsorption step. Figure 1B presents the fit of the first adsorption step of the obtained isotherm, while Table 1 summarizes the calculated value for the utilized models. For the calculations, the minimal Residual Sum Squared (RSS) method and Solver (the Excel add-in) were applied. Langmuir model assumes the adsorption on the homogeneous adsorbent, i.e. the same binding energies of all binding sites. Additionally, the model implies the monolayer adsorption character^21^. It is also noteworthy to mention that both Langmuir and Freundlich constants express the binding affinity of the adsorbate to the adsorbent. Thus, in case of metal-protein interactions, first adsorption layer may indicate the Fe^3+^ chelation or nanoparticles formation. Instead, iron in second and all subsequent layers should bond less strongly, e. g. through electrostatic interactions. Thus, taking into account the estimated molecular weight of the bLTF utilized in the study (≈ 83 kDa by MALDI-TOF MS^25^) and q_m_ derived from isotherm it can be predicted ≈ 54 additional binding sites on the surface of the protein. However, the values of the statistical metrics, i.e. square of the correlation coefficient R^2^ and standard error S, have shown the more accurate description of the first sorption stage by Freundlich isotherm. Freundlich model belongs to the oldest and most commonly used isotherm expressions. The model assumes the heterogeneous non-ideal character of the adsorption process. Moreover, the model is not restricted to monolayer formation^26^. Finally, comparing the obtained data of maximum adsorption and Langmuir constant for Fe^3+^ binding to bLTF (36.29 mg/g and 0.08 L/mg) with the data of Zn^2+^ binding to Ova-albumin (10.97 mg/g and 1.95 L/mg^23^) and β-lactoglobulin (104.40 mg/g and 0.01 L/mg^27^) it may be assumed the formation of a homogeneous Fe-bLTF complex, but not the formation of nanoparticles. In the case of Ova-albumin, the relatively high Langmuir constant can be explained by the formation of Zn/ZnO nanoparticles.

**Table 1:** The calculated values derived from Freundlich and Langmuir isotherm models.

Freundlich isotherm				Langmuir isotherm			
K_F_ [mg/g]	1/n	S	R^2^	K_L_ [L/mg]	q_m_[mg/g]	S	R^2^
4.956	0.454	2.7·10^−6^	0.9994	0.080	36.29	18.1	0.9770

Additionally, the changes of Gibbs free energy ΔG of iron binding to protein were calculated. The determined ΔG for the first adsorption stage gradually decreased depending on the concentration of the utilized Fe^3+^ and was in the range from − 24.82 to − 15.40 kJ/mol (the shown values represent Gibbs free energy observed for the lowest and highest Fe^3+^ concentrations). The results indicate the spontaneous nature of the sorption process and the heterogeneous character of the protein surface in terms of binding energies. ΔG values for the second stage were in the range from − 15.74 to − 13.90 kJ/mol and for the last concentration point (the beginning of the third adsorption stage) equals − 14.59 kJ/mol. The calculated values indicate that the majority of iron should bond loosely to the protein and do not induce the formation of new metal-binding sites. The binding energies of metals in structurally defined metal-binding sites such as active sites of an enzymes or zinc fingers usually are much higher. For instance, F. Bou-Abdallah and T. Giffune have performed the measurements of binding energies for Fe^2+^ and Zn^2+^ to apo-human transferrin by isothermal titration calorimetry (ITC)^28^. The determined ΔG depending on utilized conditions was in the range from nearly − 171 to − 195 kJ/mol which is comparable to the energies of some covalent bonds. The affinity of Fe^3+^ to transferrins has shown to be even higher^4^ than Fe^2+^ or Zn^2+^, thus the binding energyfor the respective specie should also be higher.Moreover, it is worth noting that the calculated in our studies values represent the average binding energies for several ions. Thus, for the differentiation of each particular iron ion, there is a need to perform additional studies in the future.

Still, it should be noted that Fe^3+^ in complexes more often have octahedral geometry and can form multinuclear coordination compounds. Iron in the proteins also can appear in the multinuclear form. For instance, the hydroxylase protein of *Methylococcuscapsulatus* has a dinuclear iron center at the active site^29^. The connections between iron atoms are made with oxygen-containing ligands, e.g. hydroxide, glutamic acid, acetate. The very extreme case of the Fe-rich protein complexes can be illustrated by unique Fe-storage protein—ferritin. The form of the iron in the ferritin depends on the number of bounded Fe^3+^^30^. When a small amount of Fe^3+^ is bound to ferritin they are coordinated to oxygen-containing groups. With the increased amount of bounded iron, the inorganic Fe-containing core with the crystal structure of ferrihydrite forms inside the ferritin cavity. Interestingly, Ghosh et al. have observed the formation of iron nanomineralization within fibrillation of holo human serum transferrin^31^. The iron mineral cores were shown to have a similar form which is ordinarily accumulated within ferritin^32^. Thus, such structures may also form in Fe-bLTF complexes as all transferrins have highly homologous structures and reveal similar properties. The authors also suggested that such the spontaneous processes involving transferrins may occur in the tissues and can lead to neurodegenerative disorders, but additional studies should be provided. However, the goal of our study was to obtain a homogeneous Fe-rich protein complex, which can be utilized as food additives. Thus, the synthesis of the Fe-bLTF complex for further investigations was performed with the utilization of Fe^3+^ solution with a concentration of 600 mg/L. The excess of the iron was utilized to obtain complex with fully filled monolayer and with maximum yield.

### Molecular docking

3D structure of bLTF downloaded from rcsb website contains particles that interfere MIB analysis. Thus, using Molegro Molecular Viewer 7 all particles of water and cofactors were removed. The MIB algorithm compares the query of protein structure with metal-binding template in the database and shows the results of fit by the score where the highest score indicates the higher probability of the binding^33^. The molecular docking analysis within MIB showed as many as 32 potential binding sites for Fe^3+^ interaction with bLTF (Table S. 1.). The most common amino acid residue taking part in the Fe^3+^ was cysteine (14). Despite the indication of cysteine as the amino acid that most frequently interacts with the Fe^3+^, the result for this binding does not reach the highest scores. Instead, the best results were obtained for aspartic acid (7.844), histidine (7.844) and tyrosine (7.844). The Table S. 2. shows the remaining amino acids with the calculated Score value and the number of occurrences. The obtained results are consistent with literature data which indicate that residues containing carboxyl and hydroxyl groups form the strongest bonds with Fe^3+^^34^. Moreover, from 32 predicted binding sites 6 combinations correspond to the previously described iron-binding sites in N- and C- lobes^4,17^ of bLTF which confirms the usefulness of the utilized algorithm. Still, it was shown that at least 26 additional combinations of amino acid residues can form Fe^3+^-binding sites in bLTF. The graphical presentation some of them can be find on Fig. S. 4. The obtained results indicate that MIB is a fast and simple tool for the prediction of metal-binding sites. However, it has lots of limitations as the prediction is made only within the templates included in the database which may be not enough for the determination of all possible binding sites. Moreover, water molecules are indispensable part of such kind of interactions^23,25^ and should not be omitted in molecular docking analysis as it can change dramatically the final result. Thus, more sophisticated calculations should be performed in the future.

### Characterization of Fe-bLTF complex

The synthesized complex was subjected to ICP-MS analysis for iron content quantification. The adsorption capacity of bLTF was determined as 71.37 ± 1.53 mg/g when Fe^3+^ solution with a concentration of 600 mg/L was utilized. However, the complex synthesis procedure, among others, comprises a double washing step, which was used to remove iron excess.Thus, the resulting complex contained only 32.97 ± 4.41 mg/g of iron which equals to ≈ 50 iron ions per one protein molecule. Previous studies have revealed that at unfavorable conditions, when the metal-protein complex formation did not occur the as performed washing procedure was enough to eliminate non-bonded and loosely-bonded metal from protein^25^. Interestingly, the maximum adsorption capacity calculated from the Langmuir model equals 36.29 mg/g. The results may indicate, that the first adsorption layer was fully filled with iron in the obtained complex. Moreover, the Fe^3+^ form relatively strong bonds with the protein in this layer, as it remains in the complex even after washing steps.

**Figure 2:**
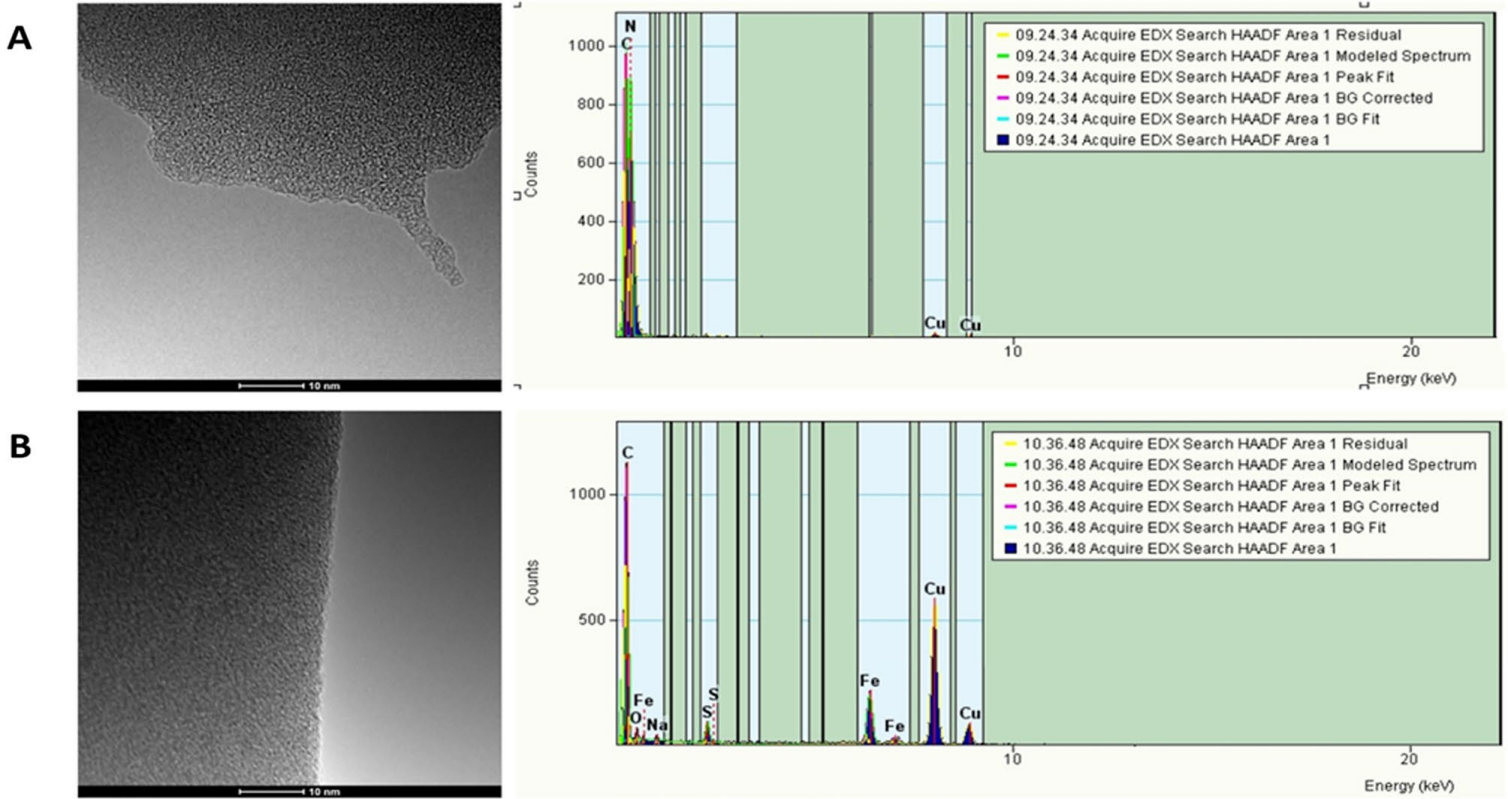
The TEM images and EDX spectra of (**A**) native bLTF and (**B**) Fe-bLTF complex.

TEM images of the synthesized complexes have revealed the formation of a homogeneous metal-protein complex, predicted from isotherm study (Fig. 2). In addition, the utilization of EDX within electron microscopy enabled the detection of an increase in Fe content in the protein. The Fe atoms were not possible to detect in the native protein, while in the EDX spectrum of the Fe-bLTF complex the appearance of peaks corresponding to Fe was observed.

**Figure 3:**
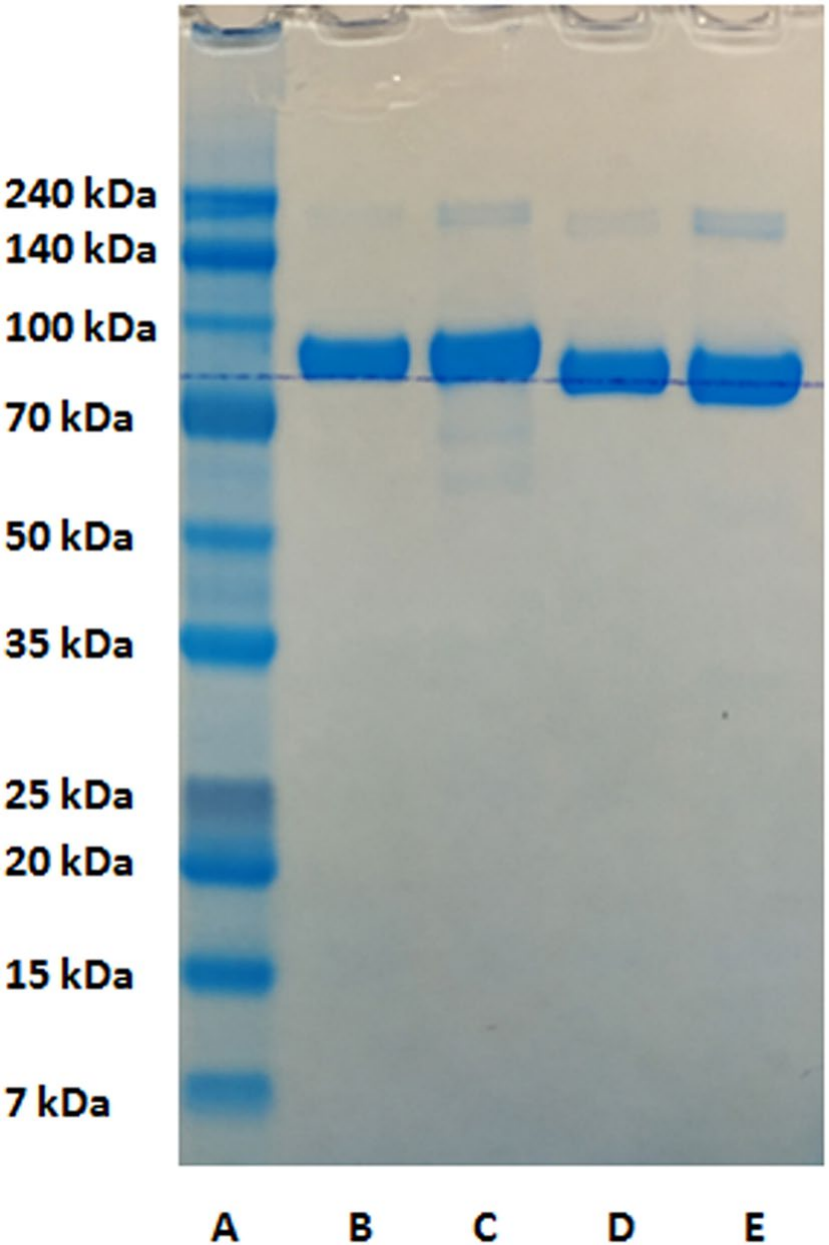
SDS-PAGE of the Fe-bLTF complex, where (**A**)—protein markers, (**B**) and (**C**)—native bLTF and Fe-bLTF complex prepared in reduced mode, (**D**) and (**E**)—native bLTF and Fe-bLTF complex prepared in non-reduced mode. The full-size electropherogram is presented in Fig. S. 4 of supplementary materials.

The SDS-PAGE analysis of obtained complex was performed (Fig. 3). The previous study performed by PAGE-IEF for different forms of bLTF has revealed changes in electrophoretic mobility of iron-saturated protein in comparison to its apo-form^35^. The present study has shown that changes in electrophoretic mobility of Fe-bLTF were observed in the case when non-reducing SDS-PAGE was utilized. The proteins’ velocity increased slightly which may indicate the formation of a more compact structure after Fe^3+^ binding^36^. Instead, the addition of a reducing agent causes the breaking of disulfide bonds and loss of the globular structure of the protein and thus should influence the metal binding ability. Consequently, no difference in bands position for bLTF and Fe-bLTF was observed in reduced SDS-PAGE, but the higher position of the bands in comparison to the non-reduced mode.

Interestingly, after the interaction of the protein with iron, the increase in the intensity of the bands between 140 and 240 kDa in both non-reduced and reduced mode was observed. Moreover, in reduced mode the bands between 50 and 70 kDa have appeared indicating partial degradation of bLTF. The results indicate the impact of Fe^3+^ on the formation of protein oligomers. Protein aggregation and fibrillation are the topics of numerous investigations. Protein fibrillation and oligomerization are considered as the leading cause of neurodegenerative disorders such as Alzheimer’s and Parkinson's diseases. The process is suggested to be connected with the environmental factors, where the imbalance of metal ion homeostasis is one of the significant factors^37^. Forced transferrin fibrillation can be performed with the utilization of dithiothreitol^38^. The method was developed for the production of nanoparticles from transferrins. Such nanoparticles are of high interest as they can be utilized as drug carriers. Dithiothreitol is the reducing agent, which breaks the S–S bonds in the protein with the formation of thiol (or sulfhydryl) groups. Then, the formation of protein oligomers can occur via chaotic uncontrolled interactions between monomers, where the rebuilding of disulfide bridges may play the main role in the aggregation process. However, the present study indicates that an increase in the dimer content rather has non-disulfide bonding nature as in other cases the intensification of dimer band would have differed for reduced and non-reduced SDS-PAGE modes.

The occurred changes can be explained by the utilized conditions, i.e. the reaction mixture comprised not only bLTF and Fe^3+^ but also a small amount of citrate ions (the iron ammonium citrate salt was utilized as a source of Fe^3+^). Citrate is well known mild reducing and stabilizing agent, which is often utilized in metallic nanoparticles synthesis^39^. It was shown that iron in the presence of citrate ions can undergo reversible reduction from Fe^3+^ to Fe^2+^^40^. During reversible metal reduction, the formation of superoxide occurs^41^, which belongs to the so-called “reactive oxygen species" (ROS). The ability of iron to promote superoxide formation is considered as one of the mechanisms of its toxicity. ROS cause the oxidation of biomolecules with the appearance of more active functional groups (e.g. peroxide or formyl) and thus can lead to the formation of protein oligomers. Moreover, the Fe^3+^ can form cross-links between protein monomers^37^. The oxidation process also can lead to protein degradation, which should be the reason for the protein degradation seen on the reducing SDS-PAGE. Thus, obtained results may indicate one of the possible mechanisms of iron's impact on the development of neurodegenerative disorders. For instance, S. Ghosh et al. have investigated the spontaneous process of human serum transferrin fibrillation^31^. The authors claim that the aggregation process can occur even after relatively small changes in the protein structure (e.g., change in the relative orientation of two lobes). From the study, it can be deduced that protein fibrillation is more extensively occur from aged protein solutions. Further results of this group were presented in Booyjzsen et al. work^32^ where the fibrillation process for recombinant human serum transferrin was presented. The study revealed that the formation of fibrils more often occurs in protein with higher content of dimers. While, it should be noted that iron in iron overload diseases can present as non-transferrin complexes consisting of iron, citrate, and BSA. Such complexes may lead to the formation of bLTF oligomers with subsequent fibrillation.

**Figure 4:**
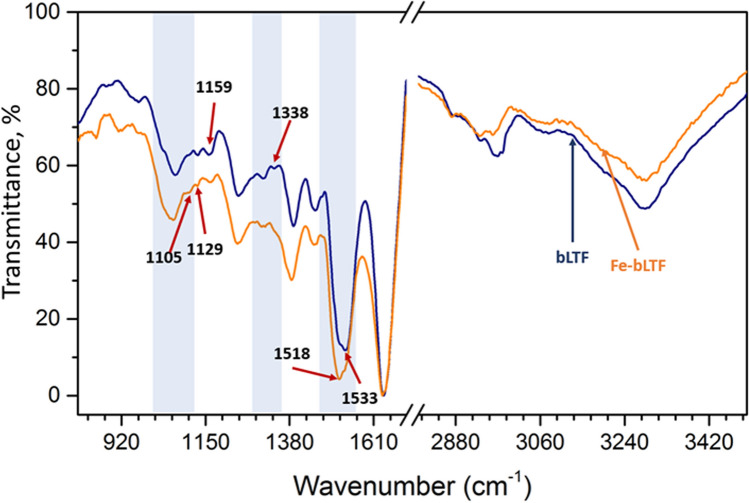
ATR-FTIR spectra of native bLTF (blue) and Fe-bLTF complex (orange), where the regions with the most significant changes are shadowed.

The FTIR analysis was performed to distinguish the functional groups which may take part in the metal binding to protein (Fig. 4). In the characteristic region for stretching vibrations of hydrogen-containing groups (2800–3700 cm^−1^) the wide absorption band with a maximum at 3280 cm^−1^ was assigned to vibrations of O–H and N–H involved in the hydrogen bonds. Symmetric and asymmetric str. vibrations CH_3_- and CH_2_-groups on spectra of native bLTF are represented by doublets at 3078/2980 cm^−1^ and 2936/2886 cm^−1^, respectively^42^. The redshifts were observed for the corresponding bands after Fe^3+^ binding, indicating the weakening of the C-H bond. The effect may be connected to the interaction between the CH group with a metal cation. For instance, it was shown that such reactions can occur between alkali metals and hexane in gas-phase, which resulted in red-shifts of corresponding bands on the FTIR spectra^43^. However, more evidently the changes in the distribution of electron densities in protein molecules after iron binding to other functional groups, e.g. carboxy, hydroxy, amino, etc. may result in such shifts. In the fingerprint region, two additional bands 1472 cm^−1^ (almost undetectable) and 1451 cm^−1^of δ_as_(CH_3_) and δ(CH_2_)vibrations can be distinguished^42,44^. The band at 1637 cm^−1^ was assigned to amide I vibrations, while the amide II band was observed at 1533 cm^−1^^44,45^. However, the same region is characteristic to the vibrations of COO^−^ group. The changes in peak maximum distribution from 1533 to 1518 cm^−1^ should indicate the interaction of Fe^3+^ through carboxylic groups of glutamic and aspartic acids, but also can be a reason of changes in protein tertiary structure. The data is constituent with the molecular docking analysis which indicates the highest probability for Fe^3+^ binding to glutamic acid^46^. Interestingly, the position of amide I is sensitive to the protein secondary structure and its position indicates the high impact of β-sheet structures in the bLTF.

More precisely secondary structure can be outlined on amide III modes, which mainly come from CN str. and NH bending (≈30% each)^44,46^. The spectrum of native protein has bands at 1338 cm^−1^, 1307 cm^−1^ distinctive for α-helix, and a more intense band at 1240 cm^−1^ of β-sheet, which is complementary to the observed position of the amide I band. After Fe^3+^ binding to protein, the shift from 1240to 1246 cm^−1^ was observed, indicating the changes in protein tertiary structure. Instead, the band at 1337 cm^−1^ has disappeared which may be due to the broadening and shift of adsorption band ν_s_(COO^−^) of glutamic and aspartic acids. This band can shift significantly upon cation chelation (+ 60/− 90 cm^−1^)^44^. The maximum of the corresponding band shifted from 1391 to 1385 cm^−1^ after iron incorporation. The data indicate, that iron rather interacts with bLTF through carboxy groups. The vibrational bands ν(C–O) of serine, aspartic, and glutamic acid appeared at 1159 cm^−1^ and 1129 cm^−1^ on a spectrum of native bLTF. Moreover, band 967 cm^−1^could also arise from the serine residue (ν(CO) or ν(CC)), while the band at 1066 cm^−1^ comes from ν(C–O) of threonine^44^. The spectrum of Fe-bLTF revealed a decrease in the 1129 cm^-1^ band, which become almost undetectable. Instead, the band at 1105 cm^−1^ has appeared, which can be assigned to ν(CN) of histidine. The shift from 1066 to 1062 cm^−1^ and broadening of the band also indicates the possible interaction of Fe^3+^ with hydroxo groups of threonine residues. However, it is noteworthy to mention that bLTF is a glycoprotein, thus in the region from 950 to 1200 cm^−1^ the bands which come from vibrations of ν(C–O), ν_st_(C–O), ν_st_(C–C) of carbohydrates can occur^47^. Moreover, bands at 914 cm^−1^ and 850 cm^−1^ that become visible after Fe^3+^ adsorption onto bLTF may correspond to *ν*CO + *δ*CCH + *ν*_asy_ (ring of pyranose) and *ν*CC + *δ*CCH + *δ*CH(β-pyranose). Generally, the appearance of the bands corresponding to more than one functional group in the same region is the main drawback of spectroscopy in the infrared range. Thus, makes it difficult to outline precisely which functional groups take part in the protein reactions.

**Figure 5:**
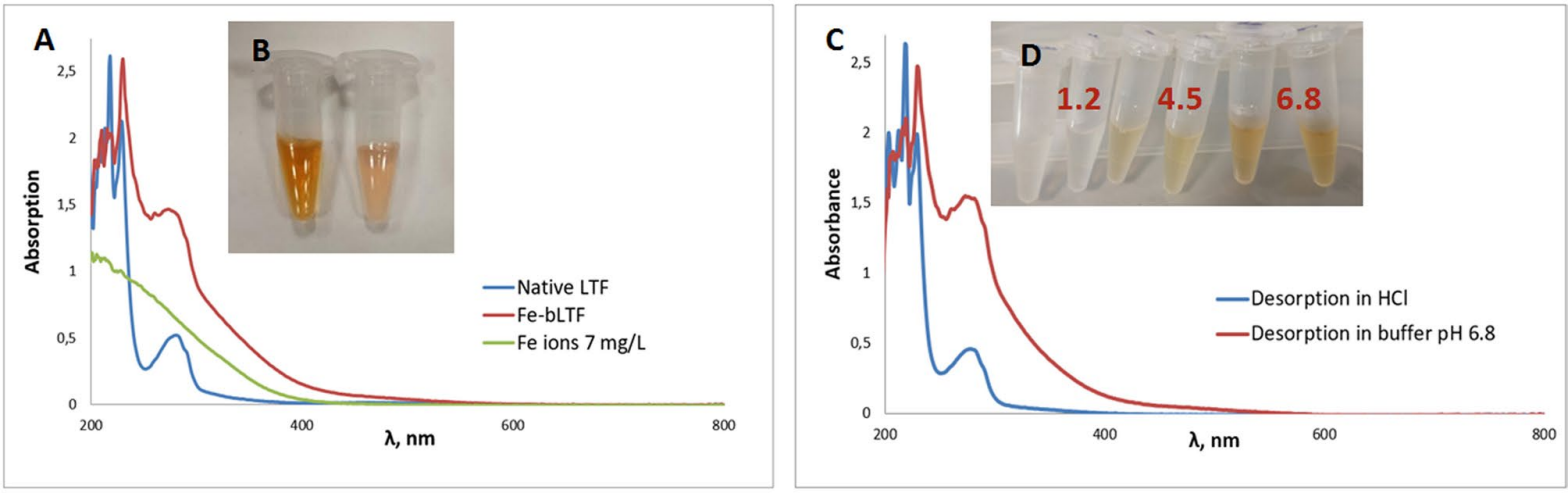
UV–ViS study of Fe-bLTF complexes: (**A**) spectra of native bLTF and Fe-bLTF complexes; (**B**) the solutions of Fe-bLTF complex on the right and unmodified bLTF on the left; (**C**) spectra of Fe-bLTF complexes after Fe^3+^ desorption in phosphate buffer pH 6.8 and 0.1 M hydrochloric acid; (**D**) Fe-bLTF complex solutions after 2 h desorption in 0.1 M hydrochloric acid (pH 1.2) and phosphate buffers pH 4.5, 6.8.

The metal complexation also can be investigated by UV–Vis spectroscopy (Fig. 5A). The nature of the light absorbance in complexes is related to d–d and charge transfer transitions. Such transitions are the reason for the development of pink-salmon color in bLTF upon Fe^3+^ embedding to iron-binding sites. The solution of unmodified bLTF revealed the intence pink-salmon color indicating the high iron conten in the protein. Further enrichment of the bLTF with iron changes the color of the complex solution to red–brown (Fig. 5B). The d–d transitions result in a weak absorption band. In the case of Fe-bLTF, the band of d–d transition can be determined at ≈ 466 nm where after Fe^3+^ adsorption onto bLTF the slight increase of the light absorption, e.g. from 0.015 to 0.058, has been observed. More intense light absorption is connected to ligand-to-metal charge transfer and was detected at ≈ 280 nm^48^. Here, the increase of absorption from 0.52 to 1.436 has occurred after Fe^3+^ binding to bLTF which indicates the involvement of tyrosines and tryptophans in metal-protein interactions.

The UV–Vis spectroscopy is indicative of the quantity of bonded iron and thus can be utilized for the investigation of the sorption process. For instance, a methodology for the determination of bLTF saturation with iron was proposed by Majka et al.^49^. The UV-difference spectroscopy was also useful for the study of preferential binding of certain metal ions to Ova-transferrin^50^. However, it rather will not be useful for the determination of bLTF iron supersaturation, e.g. additional studies should be performed to outline the regions with linear correlation.

The changes in protein color and absorbance in UV–VIS were helpful for the control of the Fe^3+^ desorption process (Fig. 5C). Iron desorption was performed in three different buffers, namely 0.1 M hydrochloric acid (pH 1.2) and phosphate buffers with pH 4.5 and 6.8. The respective buffers usually are utilized in pharmacies for the imitation of conditions in a different part of the digestive tract. After 2 h of the desorption process, the changes in the protein color have occurred at pH 4.5 and 1.2—it becomes less intense in the first case and disappeared in the second case (Fig. 5D). We were able to observe the respective changes in the UV–Vis spectra of protein after iron desorption at pH 1.2. The absorbance intensity of protein after desorption at pH 6.8 has not changed. Instead, at pH 4.5 the formation of iron phosphate precipitate has occurred which disturbs both the UV–Vis and ICP-MS analysis of the process.

The ICP-MS quantification has revealed that at pH 6.8 Fe-bLTF released only 1.08 ± 0.26% of iron which is on the level of allowed measurement error, indicating the formation of a relatively stable complex. Instead, the acidification of the solution promotes iron release. The iron desorption at pH 1.2 was estimated as 57.97 ± 3.48%. Our previous study revealed that iron release also can be promoted by the addition of other chelating agents or competing ions. The presence of citrate and zinc ions caused the iron release from holo-lactoferrin even in the basic environment of pH 7.4 and 8.6^25^. It is worthy to mention, that the intraluminal pH of the duodenum normally is near 6 which means that majority of Fe^3+^ may remain bonded in the Fe-bLTF complex in such conditions. However, the presence of other components in the food can promote iron release from the complex. Thus, further studies should be performed for the assessment of the stability of Fe-bLTF complexes and thus iron bioavailability from such preparations.

### Cytotoxicity study of bLTF

**Figure 6:**
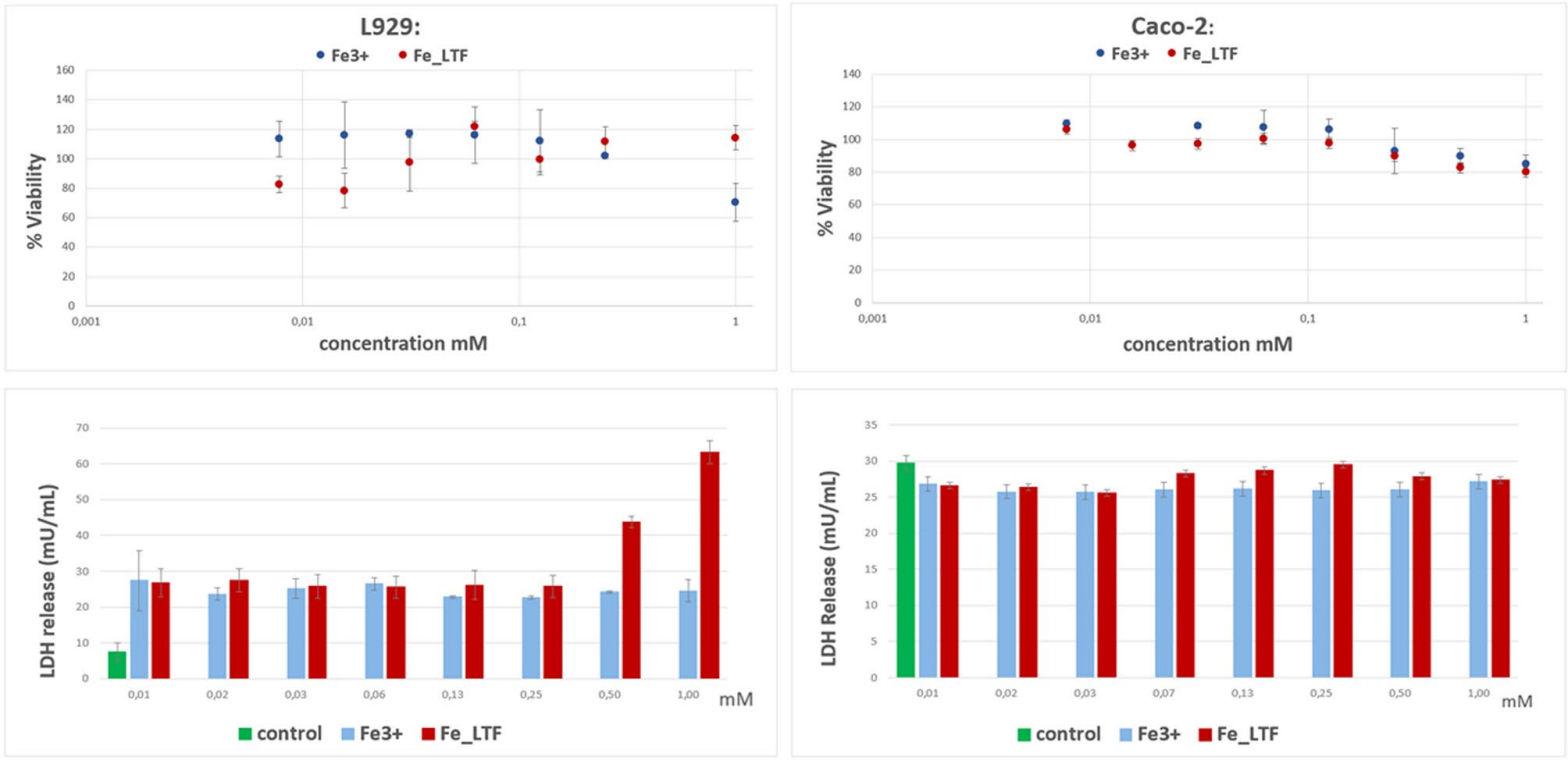
Cytotoxic effect of iron citrate and complex of Fe-bLTF in L929 and Caco-2 cells detected with the MTT reduction assay (values are expressed as a percentage of cell viability) and LDH release assay (values are expressed in mU/mL).

The comparison between cytotoxicity of Fe-bLTF with iron citrate was performed at the same iron concentration in mM (determined by ICP-MS). For studies, the L929 cell line and Caco-2 cell line were selected. L929 is a normal murine fibroblast-derived cell line which is recommended by ISO norms (EN ISO 10,993–5, ISO 10,993–12) for the biological evaluation of new medical devices. Caco-2 is a cell line derived from human caucasian colon adenocarcinoma and shows great similarity to enterocytes—intestinal absorptive cells. The results for cytotoxicity evaluation are presented in Fig. 6.

Our investigation has revealed that both iron forms, i.e. complexed with bLTF and iron citrate, at the concentration of up to 0.5 mM did not decrease the viability of the Caco-2 and L929 lines in MTT test (Fig. 6). On the other hand, the concentration of 1 mM caused a slight decrease in the viability of L929 cells to ca. 70%. Similar results were obtained for iron chloride on BALB/c and HepG2 cells. Line HepG2 was more sensitive to iron chloride than BALB/c and its viability at concentration 1 mM was ca. 60%. In studies of He et al.^51^ viability of Caco-2 cells was not limited by the concentration of iron chloride to 1 mM. Although the lower concentrations did not show a decrease in viability of L929 cells, the LDH test has shown an increase in the permeability of cell membranes similar to BALB/c and HepG2 when treated with iron chloride^52^. This phenomenon was not observed for Caco-2 cells. Both, iron complexed with bLTF and citrate did not increase the level of released LDH what means that did not adversely affect the integrity of the membranes.

Summing up, the synthesized Fe-bLTF complex does not have more negative effects on cells than the commonly used supplement of iron. In subsequent studies, it will be necessary to analyze the bioavailability of the obtained complex across the intestinal barrier and its potential with lactoferrin receptor binding. It seems that due to the presence of LTF receptors on the surface of Caco-2 cells, even small amounts of the complex should promote the absorbance of iron from the environment and improve health indicators in iron deficiency disorders.

In conclusion, we have synthesized the iron-rich Fe-bLTF complex. The batch sorption analysis was shown to be useful for the description of metal binding processes. Moreover, the derived sorption parameters can be utilized for the selection of the synthesis conditions and the prediction of final product properties. The study also revealed that during the synthesis some amount of protein can undergo oxidation and oligomerization. Moreover, the changes in protein structure with formation of more compact arrangement was observed. The synthesized complex did not release Fe^3+^ at pH 6.8, common for the duodenum. Additionally, the cytotoxicity study has shown that obtained complex at the concentration of Fe^3+^ up to 0.5 mM did not decrease the viability of the Caco-2 and L929 lines. The toxicity of the Fe-bLTF complex was not higher than the toxicity of iron citrate. Thus, the synthesized complex potentially can be used as dietary supplement in treatment of IDA.

## Materials and methods

### Chemicals and materials

Sigma-Aldrich (Steinheim, Germany) has supplied the following chemicals and materials: lactoferrin from bovine milk (bLTF), ammonium iron (III) citrate, sodium chloride, sodium hydroxide, MS-grade nitric acid, hydrochloric acid, potassium dihydrogen phosphate, Amicon^®^ Ultra Centrifugal membrane filters, ICP multi-element standard solution IV and Scandium standard solution for ICP, trypsin, fetal bovine serum (FBS), Dulbecco’s Modified Eagle’s Medium (DMEM), phosphate-buffered saline (PBS), EDTA, penicillin–streptomycin solution, L929 normal mouse fibroblast cells and Caco-2 cells, MTT assay kit and LDH release assay kit. Invitrogen Bolt™ 4–12% Bis–Tris Plus polyacrylamide gel 12 wells, Simply Blue™ Safe Stain (Coomassie G_250_ stain), MES running buffer, Load sample buffer, and Sample reducing agent were obtained from TermoFisher Scientific (Waltham, MA, USA). Perfect™ Color Protein Ladder was from EUR_X_ Sp. z o.o. (Gdansk, Poland). The set of automatic pipettes and laboratory plastics were obtained from Eppendorf (Hamburg, Germany). Moreover, deionized water was obtained with Milli-Q RG system from Millipore (Millipore Intertech, Bedford, MA, USA).

### Methods

#### *Preparation of Fe*^*3*+^*and protein solutions for the batch sorption study and complex synthesis*

To simplify the description of possible processes that may take place during interaction as the reaction medium 0.09% NaCl was utilized. The presence of salt was utilized to improve protein solubility and stability, while NaCl has shown to have no pH dependence of the preferential interaction^53^. bLTF was suspended in 0.09% NaCl solution to concentration of 5 mg/mL. For the study, bLTF standard purchased from Sigma-Aldrich (Steinheim, Germany) was utilized. Before the study, the physicochemical characterization of the bLTF was performed, among others, the iron content was determined. The results are presented in the O. Pryshchepa et al. (2022) article^1^. The bLTF from the same batch was utilized in both studies. Iron stock solution with a concentration of ≈ 6 g/L was prepared from ammonium iron (III) citrate by dissolution in deionized water. Subsequently, solutions containing appropriate Fe^3+^ concentrations were prepared from stock solution by dilution with 0.09% NaCl. The pH of all prepared solutions was adjusted to 7.4 with 0.1 M sodium hydroxide or 0.1 M hydrochloric acid.

#### *Isotherm study of Fe*^*3*+^*adsorption*

0.2 mL of bLTF suspension was transferred to a sample unit of Amicon^®^ Ultra Centrifugal Filtres cut-off 3 kDa. Subsequently, 0.2 mL of Fe^3+^ solution with a concentration of ≈ 5, 10, 20, 30, 50, 100, 200, 300, 600, 800, 1100, 1400, and 1600 mg/L (the exact concentration was determined by ICP-MS) was added to the sample unit and obtained mixture was incubated at room temperature (T ≈ 23 °C) for 24 h. After the desired time has elapsed, the reaction was terminated by centrifugation (RT, 15 000 rpm, 5 min).The filtrate was subjected to ICP-MS analysis. The analyses were performed in triplicates. Here and later the dilutions for ICP-MS analyses were performed with 1% HNO_3_ and. The analysis was performed on Shimadzu ICP-MS 2030 (Kyoto, Japan) with scandium as an internal standard. The calculations ofsorption parameters were performed as described in Pomastowski et al. and Pryshchepa et al.^22,54^.

#### Molecular docking

3D structure of diferric bLTF (code name 1BLF) was downloaded from https://www.rcsb.org website (accessed Apr 12, 2022). Experimental data snapshots for asmentioned structure were obtained by Moore, S.A. et al. with X-Ray diffraction method at 2.8 Å resolution^55^. Molegro Molecular Viewer 7 has been used to optimize the 3D structure of the protein. Metal Ion-Binding Site Prediction and Docking Server (MIB) was used for the analysis and visualization. Previously, the server was successfully used for the prediction of different metal ions binding to proteins, among others Fe^3+^ to desulfoferrodoxin from *Desulfovibriodesulfuricans* (1DFX)^33^. Sites for Fe^3+^ and bLTF interactions proposed by the program were collected in the .pdb format and further visualized in the PyMol program.

#### Synthesis of Fe-lactoferrin complex

bLTF suspension was mixed with 600 mg/L Fe^3+^ solutions at the ratio 1:1 (v/v) and incubated with stirring for 24 h (T ≈ 23 °C). The obtained complex was separated and washed twice with deionized water to remove the electrolytes excess by utilization of membrane filters cut-off 3 kDa (Amicon® Ultra Centrifugal Filters). Subsequently, the synthesized product was lyophilized. The amount of iron in the complex was determined by ICP-MS analysis. Thus, nearly 1.5 mg of the complex was mineralized with 0.1 mL of MS-Grade nitric acid. Then, first dilution was performed with deionized water. The analyses were performed in triplicates.

#### Electron microscopy

Native bLTF and Fe-bLTF complex was dispersed in ethanol, placed on a carbon-coated copper grid (Lacey Carbon Support Film 400 mesh; Electron Microscopy Sciences), and dried at room temperature. Subsequently, the samples were subjected to analysis by transmission electron microscope (TEM) (FEI Tecnai F20 X- Twin, Hillsboro, OR, USA) coupled with an EDX detector.

#### SDS-PAGE analysis

Native bLTF and Fe-bLTF complex was suspended in deionized water to a concentration of 0.5 mg/mL. The protein solutions were analyzed with the utilization of 4–12% Bis–Tris Plus polyacrylamide gel (Thermo Scientific, Waltham, MA, USA) in reduced and non-reduced mode and applying MES Running Buffer according to the standard procedure recommended by the manufacturer. Gel staining was performed with Coomassie Blue R-350 ready-to-use stain.

#### Fourier transform infrared spectroscopic (FTIR) analysis

FTIR analysis was performed for both native bLTF and modified with Fe^3+^ in the MIR range (4000–400 cm^−1^). The spectra were recorded with the utilization of attenuated total reflection (ATR) mode on Alpha FTIR spectrometer (Bruker, Billerica, Massachusetts, USA). Normalized FTIR spectra were plotted using the Origin software (v. 2015, OriginLab Corporation, Northampton, Massachusetts, USA).

#### UV–Vis spectroscopy

The UV–Vis spectra for native bLTF, for bLTF after Fe^3+^ sorption, as well as after desorption process were recorded with NanoDrop ND2000 UV–Vis spectrophotometer (TermoFisher Scientific, Waltham, MA, USA) in the range λ = 200 − 800. The spectra were plotted in MS Excel software.

#### Iron desorption study

Iron desorption tests were performed in three buffers, namely 0.1 M hydrochloric acid, phosphate buffers pH 4.5 and pH 6.8, which imitates the conditions in different parts of the digestive tract^56^. Nearly 1.5 mg of the complex was suspended in 0.5 mL of the buffer and incubated for 2 h. Subsequently, mixtures were filtrated with Amicon^®^ Ultra Centrifugal Filtres cut-off 3 kDa. The iron content in the filtrate was determined by ICP-MS analysis. The analyses were performed in triplicates.

#### Fe-bLTF cytotoxicity study

L929 cell line of mouse fibroblasts and Caco-2 cell line of Caucasian colon adenocarcinoma from European Collection of Authenticated Cell Cultures operated by Public Health England (Sigma) were cultured in DMEM supplemented with 10% (v/v) fetal bovine serum, 2 mM glutamine, and 100 U/mL penicillin, and 100 μg/mL streptomycin (Sigma). These cell lines were cultured in 75 cm^2^ flasks at 37 °C and 5% CO^2^. The cells were harvested by trypsinization using 0.25% trypsin/EDTA every 3–4 days.

For MTT and LDH release assays, cells were cultured on 96-well plates at density 2 × 10^5^ cells/mL and incubated for 24 h. Then, the medium was replaced with a new one containing Fe^3+^ of the appropriate concentration and incubated for 24 h. Then 10% (v/v) of Thiazolyl Blue Tetrazolium Bromide (MTT) solution (5 mg/mL in PBS) was added and incubated for 4 h at 37 °C. Next, the medium from wells was removed and the formazan crystals were dissolved in DMSO for 10 min by mixing. Absorbance was measured using a microplate reader (Multiskan, ThermoFisher) at 570 nm and 650 nm as background absorbance.

The LDH release assay was performed using a commercially available kit from Sigma Aldrich (Lactate Dehydrogenase Activity Assay Kit MAK066) under the standard procedure provided by the supplier. Results are presented in milliunits per mL of medium.

## Supplementary Information

Supplementary Information.

## Data Availability

All data generated or analyzed during this study are included in this published article.
