# Supplementary material for: Synthesis and physicochemical characterization of bovine lactoferrin supersaturated complex with iron (III) ions
Source: Sci Rep. 2022 Jul 26;12:12695. doi: 10.1038/s41598-022-15814-2 (PMC9325715; doi:10.1038/s41598-022-15814-2)
Supplement: Supplementary file 1 — Supplementary Information. [file 41598_2022_15814_MOESM1_ESM.pdf]

Supplementary materials

Synthesis and physicochemical characterization of bovine lactoferrin supersaturated complex with iron (III) ions.

Oleksandra Pryshchepa, Katarzyna Rafińska, Adrian Gołębiowski, Mateusz Sugajski, Gulyaim Sagandykova, Piotr Madajski, Bogusław Buszewski, Paweł Pomastowski

The amount of iron adsorbed from solution was determined using following equation:

$$q_x = (C_0 - C) \cdot V/m \quad (1)$$

where q_x is the amount of Fe^{3+} adsorbed on bLTF (mg/g), *m* is the sorbent mass (g), C_0 is the initial concentration of metal ions in aqueous solution (mg/L), *C* is the concentration of Fe^{3+} in aqueous solution after 24 h of incubation (mg/L) and *V* is the volume of solution from which sorption occurs (L).

The sorption efficiency was calculated by the equation:

$$E\ (\%) = 100 \cdot (C_0 - C)/C_0 \quad (2)$$

where *E* is the sorption effectiveness (expressed in %).

The initial concentration of metal ions in the solution as well as after sorption were calculated as follows:

$$C_x = C_y \cdot k_x \quad (3)$$

where C_x is exact value of the metal concentration in the sample, k_x is a dilution factor, and C_y is a concentration of metal solution derived from direct measurements by ICP-MS.

The metal content in the complex was calculated as follows:

$$q_n = C \cdot k\ /m \quad (4)$$

where q_n – the content of the metal in the complex, C is the concentration of the metal in the solution, derived from direct measurements by ICP-MS, k is the dilution factor.

The metal desorption was calculated according the formula:

$$X\ (\%) = 100 - (q_n - q_v) \cdot 100/qm \quad (5)$$

where X expressed in the % is the amount of desorbed metal from bLTF, qm is the metal content in the complex, qv is the amount of metal desorbed into the buffer.

The distribution coefficient (K_D) for the Fe^{3+} adsorption by bLTF was calculated based on the isotherm data at the equilibrium according to equation:

$$K_D = q_e\ /C_e \quad (6)$$

where: q_e is the amount of ions adsorbed by protein at equilibrium (mg/g), C_e is the equilibrium concentration of Fe^{3+} in solution (mg/L).

The value of the Gibbs free energy change (ΔG^0) for the silver adsorption by casein was calculated according to the following relationship:

$$\Delta G^0 = -RT \ln K_D \quad (7)$$

where ΔG^0 is the energy of adsorption in kJ/mol, R is the gas constant (8.314 J/mol·K), T is the adsorption absolute temperature in Kelvin (295K) and K_D is the dimensionless distribution coefficient.

Table S. 1. The potential binding sites of Fe^{3+} interaction with bovine lactoferrin (bLTF) calculated with Metal Ion-Binding Site Prediction and Docking Server (MIB)

No.	Binding Residues	Score
1	60D , 92Y , 192Y , 253H	2.648
2	60D , 92Y , 192Y , 253H	2.599
3	395D , 433Y , 526Y , 595H	2.49
4	395D , 433Y , 526Y , 595H	2.387
5	60D , 92Y , 192Y , 253H	2.285
6	395D , 433Y , 526Y , 595H	2.054
7	546D , 550E	1.51
8	546D , 550E	1.484
9	546D , 550E	1.472
10	546D , 550E	1.457
11	546D , 550E	1.456
12	546D , 550E	1.427
13	546D , 550E	1.414
14	546D , 550E	1.399
15	546D , 550E	1.351
16	546D , 550E	1.308
17	546D , 550E	1.3
18	433Y , 526Y	1.275
19	92Y , 192Y	1.186
20	609Q , 613H	1.043
21	115C , 157C , 198C	0.927
22	348C , 358C , 371C , 372A	0.912
23	413E , 595H	0.876
24	9C , 19C , 36C , 37V	0.868
25	157C , 198C	0.812
26	9C , 19C , 36C , 37V	0.808
27	80E , 253H	0.794
28	625C , 630C	0.787
29	625C , 630C	0.761
30	491C , 532C	0.726
31	157C , 173C	0.722
32	157C , 173C	0.712

Table S. 2. Predicted amino acid residues of bLTF that can take part in the Fe^{3+} binding, calculated with MIB

Amino Acid	Score	Number of occurrences
**ASP**	7.844/7.362/4.378	3
**HIS**	7.844/7.362/2.956	3
**TYR**	7.844/7.362	4
**GLU**	4.378/2.447/2.198	3
**GLN**	2.956	1
**CYS**	2.604/2.556/2.424/2.175/1.991/1.98	14
**ALA**	2.556	1
**VAL**	2.424	1

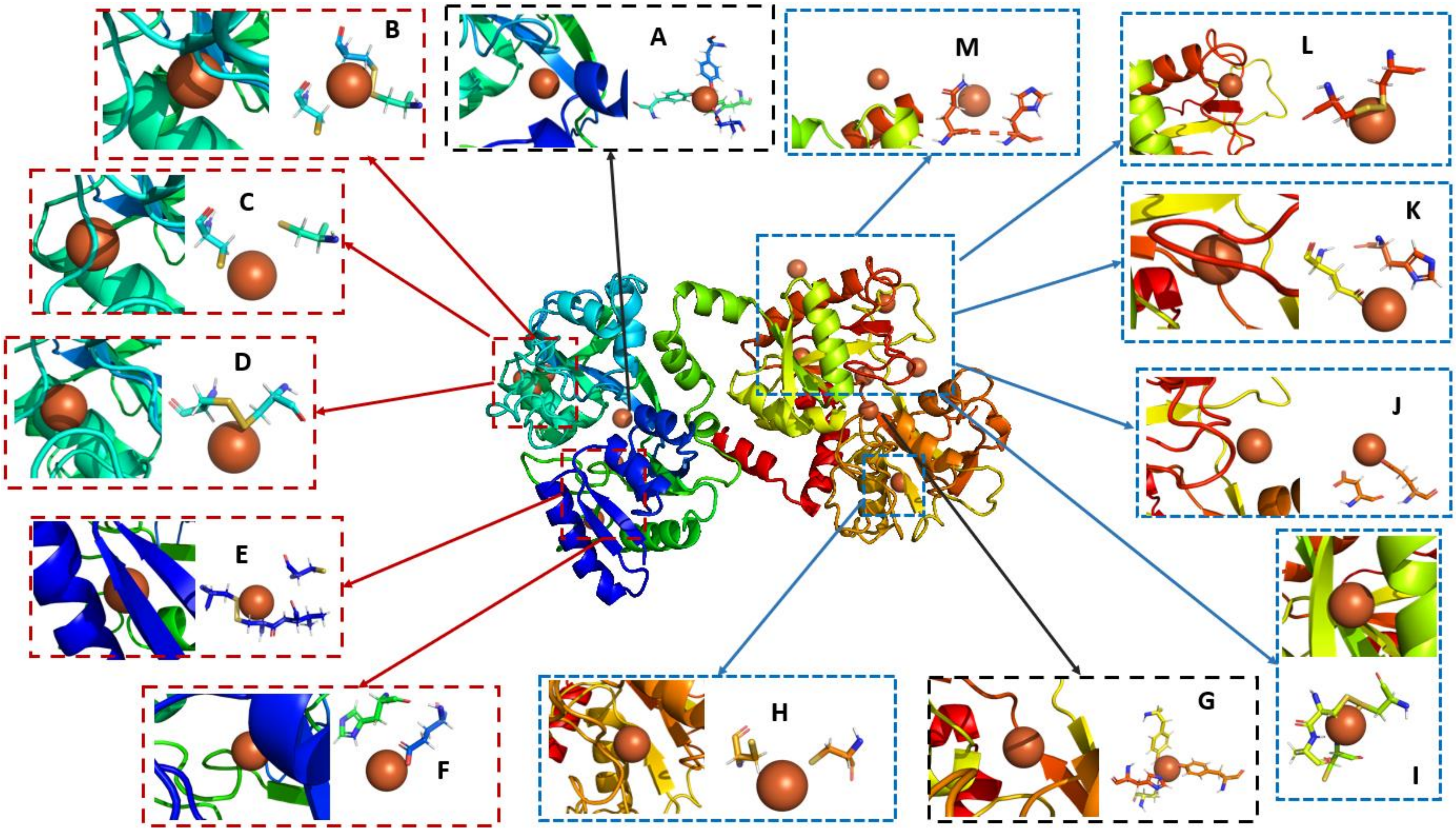

Figure S. 3. Graphical presentation of the potential Fe^{3+}-binding sites (from table S. 1) in bLTF where **A.** site No. 1; **B.** site No. 21; **C.** site No. 25; **D.** site No. 31; **E.** site No. 24; **F.** site No. 27; **G.** site No. 3; **H.** site No. 30; **I.** site No. 22; **J.** site No. 7; **K.** site No. 23; **L.** site No. 28; **M.** site No. 20.

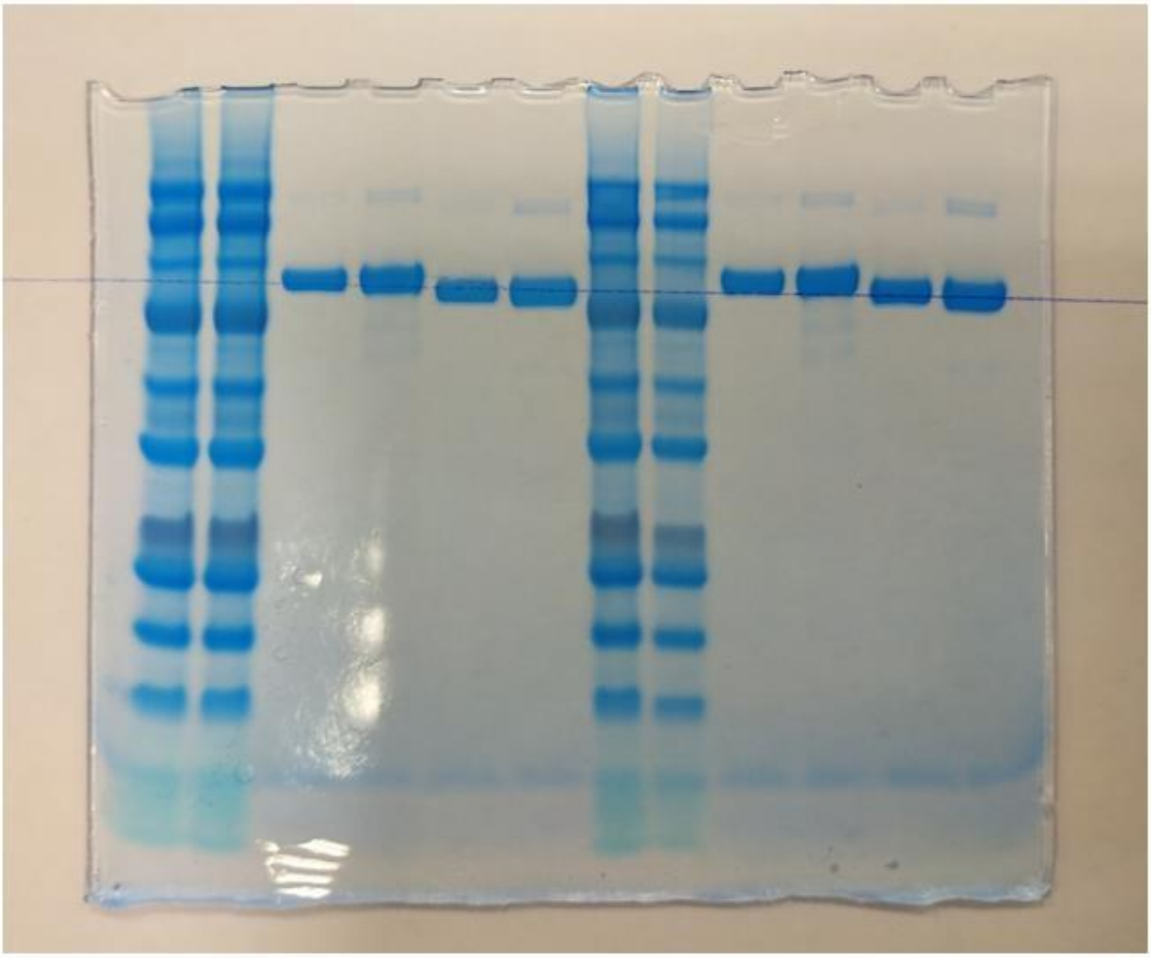

1 2 3 4 5 6 7 8 9 10 11 12

Figure S.4. The full-size (original image) electropherogram of bLTF and Fe-bLTF complex, where 1,2,7,8 – protein mass marker, 3,9 – bLTF in reduced mode, 4,10 – FebLTF in reduced mode, 5,11 – bLTF in non-reduced mode, 6,12 – bLTF in non-reduced mode.
